# Treatment-related survival patterns in diffuse intrinsic pontine glioma using a historical cohort: A report from the European Society for Pediatric Oncology DIPG/DMG Registry

**DOI:** 10.1093/noajnl/vdae155

**Published:** 2024-09-10

**Authors:** Joshua N Baugh, Sophie Veldhuijzen van Zanten, Marta Fiocco, Niclas Colditz, Marion Hoffmann, Geert O Janssens, Chiara Valentini, Darren Hargrave, Maria Wiese, André O von Bueren, Michael Karremann, Thomas Perwein, Gunther Nussbaumer, Martin Benesch, Dominik Sturm, Gerrit H Gielen, Mechthild Krause, Matthias Eyrich, Eelco W Hoving, Brigitte Bison, Dannis G van Vuurden, Christof M Kramm

**Affiliations:** Princess Máxima Center for Pediatric Oncology, Utrecht, The Netherlands; Department of Radiology and Nuclear Medicine, Erasmus University Medical Center, Rotterdam, The Netherlands; Princess Máxima Center for Pediatric Oncology, Utrecht, The Netherlands; Department of Biomedical Data Sciences, Section Medical Statistics, Leiden University Medical Center, Leiden, The Netherlands; Mathematical Institute, Leiden University, Leiden, The Netherlands; Princess Máxima Center for Pediatric Oncology, Utrecht, The Netherlands; Division of Pediatric Hematology and Oncology, University Medical Center Göttingen, Göttingen, Germany; Division of Pediatric Hematology and Oncology, University Medical Center Göttingen, Göttingen, Germany; Department of Radiation Oncology, University Medical Center Utrecht, Utrecht, The Netherlands; Princess Máxima Center for Pediatric Oncology, Utrecht, The Netherlands; National Center for Tumor Diseases, with DKFZ Heidelberg, University Hospital and Faculty of Medicine Dresden and Institute of Radiooncology - OncoRay, Helmholtz-Zentrum Dresden–Rossendorf, Dresden, Germany; Department of Radiotherapy and Radiation Oncology, Faculty of Medicine, University Hospital Carl Gustav Carus, Technische Universität Dresden, Dresden, Germany; Division of Pediatric Hematology and Oncology, University Medical Center Göttingen, Göttingen, Germany; Great Ormond Street Hospital for Children, NHS Trust London, London, UK; Division of Pediatric Hematology and Oncology, University Medical Center Göttingen, Göttingen, Germany; Division of Pediatric Hematology and Oncology, Department of Pediatrics, Gynecology and Obstetrics, University Hospital of Geneva, Geneva, Switzerland; Department of Pediatric and Adolescent Medicine, University Medical Center Mannheim, Medical Faculty Mannheim, Heidelberg University, Mannheim, Germany; Division of Pediatric Hemato-Oncology, Department of Pediatrics and Adolescent Medicine, Medical University of Graz, Graz, Austria; Division of Pediatric Hemato-Oncology, Department of Pediatrics and Adolescent Medicine, Medical University of Graz, Graz, Austria; Division of Pediatric Hemato-Oncology, Department of Pediatrics and Adolescent Medicine, Medical University of Graz, Graz, Austria; Department of Pediatric Oncology, Hematology, & Immunology, Heidelberg University Hospital, Heidelberg, Germany; Division of Pediatric Glioma Research, German Cancer Research Center (DKFZ) and German Consortium for Translational Cancer Research (DKTK), Heidelberg, Germany; Hopp Children’s Cancer Center Heidelberg (KiTZ), Heidelberg, Germany; Department of Neuropathology, University Hospital Bonn, Bonn, Germany; Department Translational Radiooncology and Clinical Radiotherapy, Helmholtz-Zentrum Dresden–Rossendorf, Dresden, Germany; German Cancer Consortium (DKTK) Dresden and German Cancer Research Center (DKFZ), Heidelberg, Germany; National Center for Tumor Diseases, with DKFZ Heidelberg, University Hospital and Faculty of Medicine Dresden and Institute of Radiooncology - OncoRay, Helmholtz-Zentrum Dresden–Rossendorf, Dresden, Germany; Department of Pediatric Hematology, Oncology, and Stem Cell Transplantation, University Hospital Würzburg, Würzburg, Germany; Department of Pediatric Neurosurgery, Utrecht University Medical Center/Wilhelmina Children’s Hospital, Utrecht, The Netherlands; Princess Máxima Center for Pediatric Oncology, Utrecht, The Netherlands; Diagnostic and Interventional Neuroradiology, Faculty of Medicine, University of Augsburg, Augsburg, Germany; Princess Máxima Center for Pediatric Oncology, Utrecht, The Netherlands; Division of Pediatric Hematology and Oncology, University Medical Center Göttingen, Göttingen, Germany

**Keywords:** diffuse intrinsic pontine glioma, diffuse midline glioma, DIPG, historical control, registry

## Abstract

**Background:**

Our aim is to investigate the association of treatment with survival in patients with diffuse intrinsic pontine glioma (DIPG) by examining 6 historical treatment paths.

**Methods:**

We retrospectively analyzed data from 409 patients with radiologically centrally reviewed DIPG, sourced from the German Society of Pediatric Oncology and Hematology HIT-HGG trial database and the SIOPE-DIPG/DMG Registry. Survival outcomes were estimated using the Kaplan–Meier method, and univariable and multivariable Cox proportional hazard models were estimated to study treatment effects.

**Results:**

The median overall survival (OS) from diagnosis was 11.2 months (95% confidence interval [CI], 10.5–11.9). Patients who by choice received no frontline treatment had an OS of 3.0 months (95% CI, 2.0–4.0), while those treated with radiation therapy (RT) alone had a median OS of 10.4 months (95% CI, 9.1–11.8). Those receiving RT combined with chemotherapy had the longest median OS of 11.7 months (95% CI, 10.8–12.6). The median post-progression survival (PPS) was 4.1 months (95% CI, 3.5–4.7). Patients who relapsed and did not receive treatment had a PPS of 2.2 months (95% CI, 1.8–2.6), while those treated with chemotherapy alone had a PPS of 4.4 months (95% CI, 3.7–5.0), and those who underwent reirradiation, with or without chemotherapy, had the longest survival after relapse of 6.6 months (95% CI, 5.3–8.0). Treatment differences remained significant in multivariable analysis adjusted for age and symptom duration in both diagnosis and relapse setting.

**Conclusions:**

This study shows increased survival outcomes associated with radiation and chemotherapy treatment or a combination thereof, at diagnosis and relapse, in a historical DIPG cohort.

## Lay Summary

Diffuse intrinsic pontine glioma (DIPG) is a very aggressive brain tumor that affects children. Different treatment approaches have been tried. In this study, the authors wanted to see how the different treatments affect how long children with DIPG live. To do this, they combined data from 409 children with DIPG. They found that children who only had radiation therapy lived for 10.4 months after their diagnosis, while those treated with both radiation and chemotherapy lived for 11.7 months. Children who did not have any treatment lived for 3 months. After the tumor got worse, children who did not receive any further treatment lived for 2.2 more months, while those treated with chemotherapy or additional radiation lived 6.6 months longer.

**Key Points**

- Without treatment, the median survival in diffuse intrinsic pontine glioma is 3 months from diagnosis and 2.2 months from relapse.
- Multimodal treatment approaches are associated with increased survival at diagnosis and relapse.
- The landmark method can correct for immortal time bias and better estimate survival after relapse.

**Importance of the Study**

We report survival in patients with no treatment, radiation only at diagnosis, and no treatment at relapse, and compare these limited treatments with more intensive treatment in both the frontline and relapse settings. This allows for comparison, although imperfect, with a more diverse set of patient outcomes. Furthermore, we show using the landmark method how to deal with immortal time bias and better estimate survival outcomes after relapse. Survival outcomes observed in our cohort provide a baseline reference value for commonly used treatment modalities. Historical control data from registry-based studies demonstrate the potential to act as external controls in innovative clinical trial designs, such as externally controlled single-arm designs, and compensate for the lack of a “standard” therapy in DIPG.

Frontline radiotherapy (RT) is standard of care in diffuse intrinsic pontine glioma (DIPG) treatment, conferring an additional 3–4 months of survival compared with no tumor-directed therapy.^1,2^ The role of systemic chemotherapy, both concomitant and adjuvant to radiotherapy, however, is a subject of debate. In the European context, 54% of treating physicians indicate using radiotherapy only and 45% combining with chemotherapy.^3^ Given many patients receive therapy beyond radiation at diagnosis, it is important to evaluate if there is a survival advantage. Furthermore, in a large series of 1100 patients with DIPG, Hoffman et al. found longer overall survival (ie, greater than 2 years) correlated with neoadjuvant and adjuvant systemic chemotherapy.^4^ This warrants further investigation into the survival benefit of additional treatments beyond standard radiation therapy (RT).

Immortal time bias poses a significant challenge in the assessment of DIPG treatments, particularly in the relapse setting. This form of selection bias is highly prevalent in observational studies published in leading journals.^5^ In such a scenario, patients are classified retrospectively using treatment status at the time of study completion, which is not known at baseline when the analysis is performed. Median overall survival (OS) is then calculated from diagnosis, regardless of the timing of therapy initiation. This erroneous inclusion of a covariate in the analysis at baseline, which is only known in the future, has the effect of underestimating the death rate in the treated group and overestimating the death rate in the untreated group. If many patients die early, as in the case of DIPG, the bias can be quite large. In our study design, we correct for immortal time bias using the landmark method.^6^

Due to the very poor prognosis of patients with DIPG, despite intensive therapeutic research efforts for the last several decades, pediatric oncologists often consider no oncological therapy an acceptable option. In the largest DIPG series published to date, 3% of patients received no oncological treatment.^4^ This may be an underestimate, as most patients were included in clinical trials. Epidemiological studies from the Netherlands and Canada report 14% and 8% of patients with DIPG, respectively, without any oncological treatment.^7,8^ The absence of untreated or minimally treated patients from observational studies inflates survival estimates toward prognostically better patients who are eligible for clinical trials.^9^

We report survival in patients with no treatment, radiation only at diagnosis, and no treatment at relapse, and compare these limited treatments with more intensive treatment. This allows for comparison with a more diverse set of patient outcomes. To conduct this project, survival outcomes were examined across 6 treatment modalities: 3 in the frontline setting and 3 at relapse. At diagnosis, modalities included (I) no treatment, (II) RT alone, and (III) RT chemotherapy. At relapse, modalities included (I) no additional treatment, (II) chemotherapy, and (III) re-RT with or without chemotherapy.

Our aim is to investigate associations with survival among these 6 “treatment paths” and estimate the effect of individual treatment modalities on survival. Prognostic information on clinical course and survival without any treatment or radiotherapy alone, versus progressive oncologic treatment, will be helpful for patients and families who are considering all available treatment options.

## Patients and Methods

### Study Population

Data were collected retrospectively on 409 patients using the German Society of Pediatric Oncology and Hematology (GPOH) HIT-HGG trial database and the European Society for Pediatric Oncology (SIOPE) DIPG/DMG Registry. The SIOPE-DIPG Registry has been reviewed, and the Medical Research Involving Human Subjects Act (WMO) does not apply (protocol reference number 22/724). This study was also approved by the IRB at University Medical Center Göttingen.

All patients had radiologically centrally reviewed DIPG, mostly not biopsied. Inclusion criteria for the SIOPE-DIPG Registry were based on protocol version 1.0. These criteria for patient inclusion included patients with DIPG, defined as a T1-weighted hypointense and T2-weighted hyperintense tumor with at least 50% involvement of the pons (DIPG) on T2, and as confirmed by expert neuroradiologists via the central radiology review procedure. Furthermore, at least one of the following typical brainstem symptoms should be present: cranial nerve deficits, long tract signs, or ataxia. The onset of symptoms should be short, preferably less than 3 months and at maximum 6 months. If the duration of symptoms was longer than 6 months, a biopsy was usually performed to confirm high-grade glioma. Nevertheless, due to the nature of a retrospective cohort, in some cases, the duration of symptoms before diagnosis was not clearly defined. All GPOH-HIT-HGG patients in the present study were trial patients and underwent confirmation of DIPG diagnosis by central neuroradiological review. Only patients between ≥3 and <18 years of age at diagnosis were included in this study. No patients were excluded based on the year of diagnosis. Patients in this cohort were treated between 1990 and 2017. Initial data collection was completed on November 27, 2019, marking the end of the follow-up period.

In the frontline “untreated” group, declining treatment was voluntary in all 20 patients. Clinical records were checked to ensure these patients did not forgo treatment after diagnosis because of rapid deterioration or poor performance/clinical condition. The “radiotherapy-only” group was either a voluntary treatment decision or based on recommended national standards at the time of diagnosis. Patients on whom we did not have reliable data on the relapse situation and/or no centrally reviewed data were excluded. Detailed treatment information on individual treatment modalities was limited in this study due to the retrospective design. Information on systemic therapy regimens is available in Supplementary Table 1.

### Statistical Analysis

The Kaplan–Meier methodology was used to analyze survival data. Median OS time was computed from date of diagnosis to death; OS is reported at 3 months, 6 months, 1 year, 2 years, and 5 years. To study the effect of different treatments upon progression, the landmark method was also used.^6,10^ A landmark point was set from the first relapse time for patients who experienced a relapse. Patients with no documented relapse were not included. Date of first relapse/progression was reported by the enrolling center and defined as first clinical or radiographic progression. Univariable and multivariable Cox proportional hazard regression models were used to quantify the effect of each treatment on survival. The covariates such as age at diagnosis and symptom duration, the well-established positive clinical prognostic factors, were incorporated into the multivariable model.^11^ Two Cox models were estimated: 1 from diagnosis and 1 from relapse. To examine differences in sex and age distribution, at baseline, *t*-test and Pearson’s chi-square tests were used. IBM SPSS Statistics versions 26 and 29 (Armonk, NY) were used to perform the analysis.

## Results

### Patient Characteristics

At baseline, among 409 patients (Table 1), the median age was 7.5 years (95% CI, 6.8–7.8) with a range of 3–17.8 years. The median age of the 3 treatment groups—(I) no treatment (5.7 years), (II) radiotherapy alone (7.0 years), and (III) radio-chemotherapy (7.7 years)—was significantly different (*P* = .05). Most patients (72 %) were between 3 and 10 years of age, and 28% were between 10 and 18 years. The sex distribution was 52% female and 48% male and not significantly different between treatment groups (0.98). The biopsy rate was 23%. Most patients had a symptom duration <6 weeks (63.3%), followed by 6–12 weeks (19.6%), 12–24 weeks (8.6%), >24 weeks (5.6%), unknown (2.9%), and not significantly different between baseline treatment groups (*P* = .10).

**Table 1.** Baseline Patient Characteristics (*n* = 409).

Characteristics	*n* (%)	*P*
Sex		.96
Male	196 (48)	
Female	213 (52)	
Age at diagnosis		
Range	3.0–17.8 y	
Median age (95% CI)	7.5 y (6.8–7.8)	.05
No treatment	5.7 y	
Radiotherapy alone	7.0 y	
Radio-chemotherapy	7.7 y	
Age by group		
3–10 years	293 (72)	
>10–18 years	116 (28)	
Biopsy		
Yes	92 (23)	
No	317 (77)	
Biopsy histology (pre-WHO 2016)		
GBM, WHO Grade IV	33 (35.9)	
AA, WHO Grade III	29 (31.5)	
Astrocytoma, NOS	10 (10.9)	
LGG	11 (12)	
Unknown	9 (9.7)	
DMG H3K27-altered	19/312^*^	
Symptom duration		.10
<6 weeks	260 (63.3)	
6–12 weeks	80 (19.6)	
12–24 weeks	35 (8.6)	
>24 weeks	23 (5.6)	
Unknown	12 (2.9)	

Abbreviations: AA= anaplastic astrocytoma; CI = confidence interval; DMG= diffuse midline glioma; GBM= glioblastoma multiforme; GPOH = German Society of Pediatric Oncology and Hematology; LGG= low grade glioma; NOS= not otherwise specified; WHO = World Health Organization.

^*^Molecular data available within the GPOH cohort only.

### Survival Outcomes

**Figure 1.**
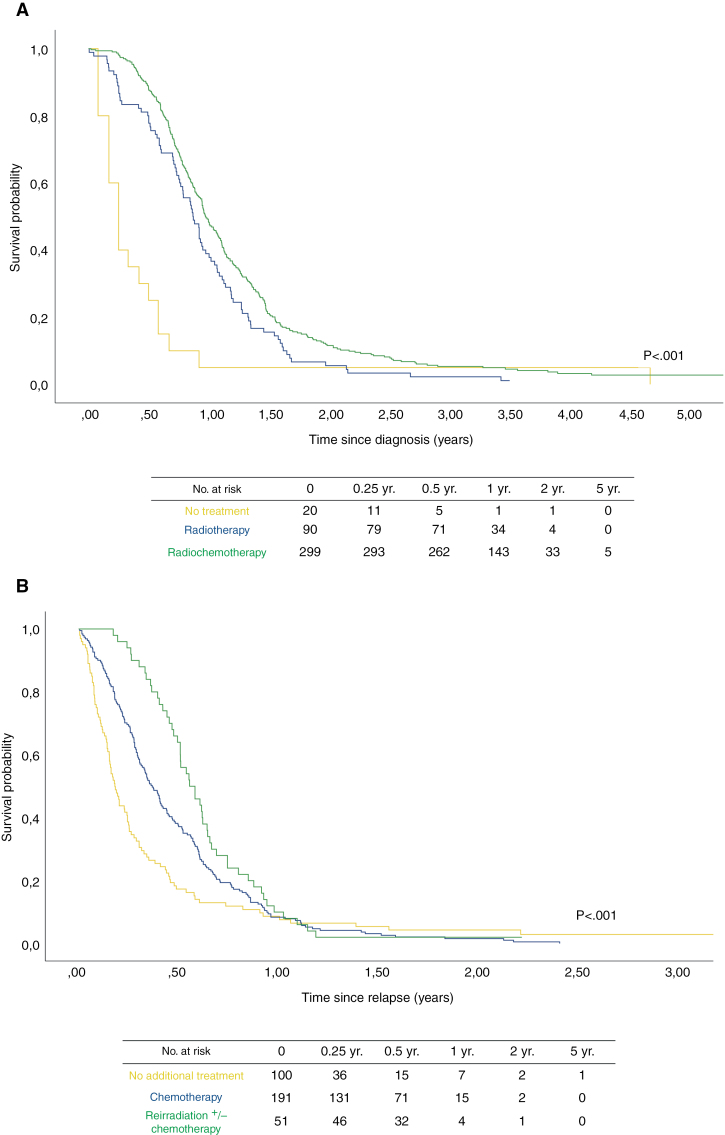
Estimated Kaplan–Meier survival time from (A) diagnosis (*n* = 409) and (B) relapse (*n* = 342).

From diagnosis, the median OS was 11.2 months (95% CI, 10.5–11.9) for the whole cohort (*n* = 409). For the different treatment groups, the median OS was 3 months (95% CI, 2.0–4.0) for patients who received no treatment, versus 10.4 months (95% CI, 9.1–11.8) for those who were treated with radiotherapy alone, and 11.7 months (95% CI, 10.8–12.6) for patients receiving radio-chemotherapy (Figure 1A, *P* < .001). For patients who received no treatment, OS at 6 months and 1 year was 25% (95% CI, 6%–44%) and 5% (95% CI, 0%–15%) respectively, in comparison with patients treated with RT only, with an OS of 80% (95% CI, 72%–88%) at 6 months and 39% (95% CI, 29%–49%) at 1 year. Radio-chemotherapy patients had an OS of 88% (95% CI, 84%–92%) at 6 months and 49% (95% CI, 43%–54%) at 1 year (Table 2).

**Table 2.** Survival Time From Diagnosis With 95% Confidence Interval

First-line treatment (*n* = 409)	Group I: None (*n* = 20)	Group II: Radiotherapy (*n* = 90)	Group III: Radio-chemotherapy (*n* = 299)
Median survival	3.0 mo. (2.0-4.0)	10.4 mo. (9.1–11.8)	11.7 mo (10.8–12.6)
** **Survival at 6 mo	25% (6%–44%)	80% (72%–88%)	88% (84%–92%)
** **Survival at 1 y	5% (0%–15%)	39% (29%–49%)	49% (43%–54%)
** **Survival at 2 y	5% (0%–15%)	6% (1%–10%)	12% (8%–15%)
** **Survival at 5 y	0%	0%	3% (1%–5%)
Median overall survival 11.2 mo (10.5–11.9)			

In patients who experienced a relapse (*n* = 342), the median post-progression survival (PPS) was 4.1 months (95% CI, 3.5–4.7). For the respective treatment groups, PPS was 2.2 months (95% CI, 1.8–2.6) for patients with no relapse treatment versus 4.4 months (95% CI, 3.7–5.0) for patients who received relapse chemotherapy, and 6.6 months (95% CI, 5.3–8.0) for patients receiving reirradiation with or without relapse chemotherapy (Figure 1B, *P* = <.001). For patients with no additional treatment after relapse, the survival at 6 months was 17% (95% CI, 10%–25%). With chemotherapy, the survival at 6 months was 37% (95% CI, 31%–44%), and for patients receiving reirradiation with or without relapse chemotherapy, the 6-month survival was 64% (95% CI, 51%–77%, Table 3). The number of events from both relapse and diagnosis is available in Supplementary Table 2.

**Table 3.** Survival Time From Relapse With 95% Confidence Interval

Relapse treatment (*n* = 342)	Group I: None (*n* = 100)	Group II: Chemotherapy (*n* = 191)	Group III: Reirradiation +/− chemotherapy (*n* = 51)
Median survival	2.2 mo (1.8–2.6)	4.4 mo (3.7–5.0)	6.6 mo (5.3–8.0)
** **Survival at 6 mo	17% (10%–25%)	37% (31%–44%)	64% (51%–77%)
** **Survival at 1 y	8% (2%–13%)	8% (4%–12%)	10% (2%–18%)
** **Survival at 2 y	4% (0.2%–8%)	1% (0%–2%)	2% (0%–6%)
** **Survival at 5 y	3% (0%–6%)	0%	0%
Median post-progression survival 4.1 mo (3.5–4.7)			

### Treatment Modalities

For patients who received no treatment and radiotherapy at diagnosis, hazard ratios (HRs) for OS of 3.65 (95% CI, 2.30–5.81) and 1.34 (95% CI, 1.05–1.70) were found, respectively, relative to radio-chemotherapy. From relapse, patients with no additional treatment had an HR for OS of 1.44 (95% CI, 1.12–1.85), and reirradiation with or without relapse chemotherapy had an HR of 0.72 (95% CI, 0.52–0.98), relative to chemotherapy.

On multivariable analysis, using the covariates of age at diagnosis and symptom duration, the adjusted hazard ratios (aHRs) from diagnosis for patients with no treatment were found to be 5.48 (95% CI, 3.29–9.14) and 1.26 (95% CI, 0.99–1.60) for patients receiving radiotherapy, relative to radio-chemotherapy. From relapse, aHRs of 1.46 (95% CI, 1.13–1.87) for untreated patients and 0.71 (95% CI, 0.51–1.0) for patients receiving reirradiation with or without relapse chemotherapy were found, relative to chemotherapy (Table 4).

**Table 4.** Estimated Univariable and Multivariable Cox Proportional Hazard Regression Models

		HR (95%CI)	aHR (95%CI)
Diagnosis	No treatment	3.65 (2.30–5.81)	5.48 (3.29–9.14)
	Radiotherapy alone	1.34 (1.05–1.70)	1.26 (0.99–1.60)
	Radio-chemotherapy^*^		
Relapse	No additional treatment	1.44 (1.12–1.85)	1.46 (1.13–1.87)
	Reirradiation +/− chemotherapy	0.72 (0.52–0.98)	0.71 (0.51–1.0)
	Chemotherapy^*^		

Hazard ratio (HR) and adjusted HR (aHR) along with 95% confidence interval (CI).

^*^Reference category (ie, largest group).

## Discussion

Although not evidenced in a randomized fashion, our data suggest an association between systemic therapy and increased survival in patients with DIPG, as observed in other large-scale assessments using historical cohorts.^4,11,12^ Our results are in line with a systematic review by Gallitto et al. encompassing all radiation regimens, in which patients with concomitant systemic therapy had an OS of 11.5 months, in comparison with 9.4 months for radiation-only patients.^13^ A beneficial role for systemic chemotherapy has recently also been supported in adult H3K27M-altered DMG.^14^ The survival benefit of irradiation in DIPG is well documented over the last 50 years, and in the absence, the disease progresses quickly.^15^ Ataç et al. documented cases of 3-week survival in children too sick to receive irradiation.^16^ We excluded patients too sick to receive therapy. However, we do report survival in patients who elect not to receive therapy and those who die very shortly after diagnosis. These patient populations are not captured in clinical trials.

Unique to our survival analysis is the inclusion of patients who received limited treatments (ie, no treatment, irradiation only at diagnosis, and no additional treatment at relapse), in comparison with more intensive treatment. This allows for comparison with a more diverse set of patient outcomes. Prior reference values for survival outcomes were largely based on small nonrandomized, mostly single-institution clinical trials.^17,18^ A note of caution must be used to compare patient outcomes in the absence of treatment, given the likely underlying differences in treated versus untreated patient populations. However, using the SIOPE and International DIPG/DMG Registries, we can better define outcome measures observed in historical cohorts, such as PPS, as described in this study.^17^ Median survival times observed in our cohort provide a baseline reference value (or historical control) for commonly used treatment modalities, from which new investigational therapies can be compared. Differences in survival, albeit small in scale, are essential to document for patients/families and clinicians alike. By knowing how long patients with DIPG survive broadly without treatment or radiotherapy alone, treating physicians have more detailed information to employ when talking to patient families.

Novel to our study design is the use of the landmark methodology, in which the date of first relapse was used as a landmark time point.^6,10^ This method controlled for immortal time bias and allowed us to better measure what survival can be attributed to relapse therapies. We used PPS, rather than OS from diagnosis to evaluate relapse treatments, and patients were grouped using only treatment information known at relapse to evaluate the effect of relapse therapies on survival. This design is both clinically relevant and easily transferable to other observational DIPG studies investigating relapse treatments.^17^ There may be a short gap between date of relapse and start of relapse treatment, but this is considered minimal in DIPG.^19^ It should be noted that progression was not centrally reviewed and therefore remains a limitation in this study.

Our survival data suggest multimodal therapy is associated with increased survival in both the primary and relapse settings. Patients receiving no frontline treatment had an increased risk of death, 3.5 times that of patients receiving radio-chemotherapy. Patients receiving radiotherapy alone had 34% increase in the expected hazard of death relative to radio-chemotherapy. At relapse, patients with no additional treatment had a 44% increase in the expected hazard of death, relative to maintenance chemotherapy alone, while reirradiation with or without relapse chemotherapy was protective, with a 28% reduction (Table 4). On multivariable analysis, adjusted for age and symptom duration, untreated patients at diagnosis had a risk of death 5.5 times that of patients receiving radio-chemotherapy. In addition, multimodal treatments remained significantly associated with increased survival on multivariable analysis. Caution is required in the interpretation of the results due to the observational retrospective nature of the data. It was also not possible for these reasons to delineate which patients received reirradiation, with or without chemotherapy, precluding a subanalysis to estimate the effect of reirradiation alone on survival.

Survival rates were higher with multimodal therapy. In the frontline setting, at 6 months and 1 year from diagnosis, 8% and 10% more patients, respectively, were alive in the radio-chemotherapy group than in the radiotherapy group (Table 2). Similarly, from relapse, at 6 months, 27% more patients in the reirradiation with or without relapse chemotherapy group were alive than in the relapse chemotherapy group (Table 3). Importantly, all survival differences in our study disappeared within 1 year after relapse, aside from a few outliers, with DIPG remaining almost uniformly fatal. Data availability and sample sizes precluded any subgroup analysis on specific drugs or protocols and were outside the scope of this study. Information on available systemic therapy regimens can be found in Supplementary Table 1. Durable response and long-term survival, as suspected, are not feasible using existing treatment modalities. Given the mostly palliative nature of current systemic therapy regimens, the use of chemotherapy must be carefully balanced with the risk of toxicity to maintain optimal quality of life.^20^

A limitation of this study is the observational and retrospective design, in which indication bias might be present. In such cases, an association between an individual treatment modality and survival could be a marker of favorable prognosis, rather than treatment efficacy.^21^ To investigate potential bias by indication, we examined age at diagnosis and symptom duration as surrogate markers of prognosis using the available data.^11^ Median age at diagnosis differed between the frontline groups, namely due to the younger age of the untreated group (median 5.7 years), relative to the median of 7.5 years across all patients. This could portend that younger patients are less likely to receive treatment. Infants were specifically excluded in this study to lessen the potential for younger age to act as a confounder.^22,23^ In addition, the vast majority (72%) of patients were between 3 and 10 years of age, minimizing the impact of older age.^24^ For symptom duration, most patients had a short symptom duration of less than 6 weeks (63%) and only 6% had a long symptom duration of greater than 24 weeks (Table 1). This suggests our cohort is a robust representation of the DIPG patient population and is comparable with other historical cohorts.^4,7,8^ To further limit the impact of confounders, a multivariable analysis was performed. Age and symptom duration were found to be confounders and were adjusted using a multivariable Cox regression. Treatment-related survival patterns as mentioned earlier remained significant.

Therapeutic efficacy has been difficult to discern in DIPG, given the heterogeneous comparisons between trials caused by inconsistent inclusion and exclusion criteria, unclear/differing endpoints, and small sample sizes.^18^ Prior to the discovery of histone mutations and a more widespread utilization of biopsy, DIPG diagnoses in clinical studies, without MRI central review, included less aggressive pediatric brainstem gliomas, artificially inflating survival estimates and largely explaining initial survival differences.^25^ Central review by MRI, as performed in this study, has been proven to eliminate low-grade gliomas and is consistent at eliminating atypical cases when performed by an experienced neuroradiologist.^12,26^ The International and SIOPE-DIPG/DMG Registries enable population-based research to be done for the first time in DIPG utilizing centrally reviewed MRI data.^27,28^

The use of chemotherapeutics in the field has not advanced substantially, over the last 3 decades, in part due to the issues mentioned earlier, but also an inability to identify and develop effective combinatorial therapies preclinically, coupled with a lack of innovation in clinical trial design.^29,30^ Recent biological discoveries dispel the idea that brain tumors are “monogenetic and monoclonal” and necessitate a holistic view of the cancer.^30,31^ Newly developed biologically and immunotherapy-driven approaches show promise in early-phase studies; however, all patients still succumb to their disease.^32–35^ Acquired therapeutic resistance, tumor heterogeneity, and drug delivery remain barriers to overcome in the development of curative therapeutics.^30,36^ For ethical reasons, single-arm clinical trials without a concurrent control arm have made up most early-phase trials in DIPG. An overreliance on single-arm trial designs has been suggested as a leading factor for the lack of successful trial development in neuro-oncology. Single-arm designs do not account for differences between populations or different standards to assess outcomes across trials, nor do they control for biases.^37^

External controlled clinical trials using historical control data offer an alternative design. The incorporation of historical controls, if comparable with trial participants, can reduce variance, increase power, and improve trial efficiency, thereby reducing the number of patients needed.^38^ External control data from the Registries can be used in the design of several externally augmented trial designs, as outlined in Polley et al., with considerations specific to pediatric brain tumors available in Margol et al.^39,40^ Perhaps the most applicable design to the current DIPG/DMG trial environment is the externally controlled single-arm design. Using an externally controlled single-arm design, statistical adjustments such as matching can be used to account for baseline differences between the historical control and experimental groups. This reduces bias in comparison with standard single-arm trials. Furthermore, during the analysis, treatment effects can be estimated directly between the experimental and external controls using patient-level data, rather than extrapolating using a published benchmark. Historical controls can also be used to inform the interim trial analysis, but these designs are still in the exploratory phase.^39,41^

In the pathway forward to cure primary brain tumors, it is required to systematically confront existing faults within the research pipeline. It entails rethinking the design of neuro-oncology clinical trials.^30,37^ Due to the lack of adequate standard treatment and the high desire for improvement of the fatal prognosis, the use of innovative designs using historical controls is ideally suited. Historical control data from registry-based studies like the present one demonstrate the potential to inform trial design and compensate for the lack of standard therapy in DIPG. Future trials will mandate biological subgrouping based on the presence of histone mutations. However, the IDIPGR and SIOPE Registries now also incorporate DMGs and capture available pathogenomic data from these patients. Historical controls in the absence of biopsy remain relevant as well. At present, 70% of pediatric patients with brainstem high-grade glioma are confirmed radiographically in the United States.^42^ A biopsy still requires a delicate and invasive surgery to be performed in a specialized center and preferably in the context of a clinical trial.^43^

## Conclusions

For the first time, in a large retrospective analysis, we show by using the landmark method how to deal with immortal time bias and provide robust estimate survival outcomes in relapse DIPG. Population-based registries, such as the SIOPE and International DIPG/DMG Registries that include trial and nontrial patients, are essential to identify patterns of response in these rare cancers. Clinical trials incorporating innovative designs in an international, multi-institutional setting are needed to finally improve the fatal prognosis. Studies like the present one, which better define survival outcomes, provide a representative historical reference point, from which new investigational therapies can be compared. More precise and representative study endpoints are key to improving DIPG trial design and efficiency.^17^ Furthermore, survival data presented here may be helpful for treating physicians communicating with patient families who are considering a clinical course without any treatment or radiotherapy alone, versus progressive oncological treatment. Future studies should strive to incorporate quality-of-life parameters and balance the extension of survival with optimal quality of life.

## Supplementary Material

vdae155_suppl_Supplementary_Materials

## Data Availability

Data will be made available upon reasonable request via the authors or the SIOPE DIPG/DMG Registry.

## References

[1] Langmoen IA, Lundar T, Storm-Mathisen I, Lie SO, Hovind KH. Management of pediatric pontine gliomas. Childs Nerv Syst. 1991;7(1):13–15. 2054800 10.1007/BF00263825

[2] Bennett J, Erker C, Lafay-Cousin L, et al Canadian pediatric neuro-oncology standards of practice. Front Oncol. 2020;10(593192):1. 33415075 10.3389/fonc.2020.593192 PMC7783450

[3] El-Khouly FE, Veldhuijzen van Zanten SEM, Santa-Maria Lopez V, et al Diagnostics and treatment of diffuse intrinsic pontine glioma: where do we stand? J Neurooncol. 2019;145(1):177–184. 31522324 10.1007/s11060-019-03287-9 PMC6775536

[4] Hoffman LM, Veldhuijzen van Zanten SEM, Colditz N, et al Clinical, radiologic, pathologic, and molecular characteristics of long-term survivors of diffuse intrinsic pontine glioma (DIPG): a collaborative report from the International and European Society for Pediatric Oncology DIPG Registries. J Clin Oncol. 2018;36(19):1963–1972. 29746225 10.1200/JCO.2017.75.9308 PMC6075859

[5] van Walraven C, Davis D, Forster AJ, Wells GA. Time-dependent bias was common in survival analyses published in leading clinical journals. J Clin Epidemiol. 2004;57(7):672–682. 15358395 10.1016/j.jclinepi.2003.12.008

[6] Anderson JR, Cain KC, Gelber RD. Analysis of survival by tumor response. J Clin Oncol. 1983;1(11):710–719. 6668489 10.1200/JCO.1983.1.11.710

[7] Veldhuijzen van Zanten SE, Jansen MH, Sanchez Aliaga E, et al A twenty-year review of diagnosing and treating children with diffuse intrinsic pontine glioma in the Netherlands. Expert Rev Anticancer Ther. 2015;15(2):157–164. 25435089 10.1586/14737140.2015.974563

[8] Fonseca A, Afzal S, Bowes L, et al Pontine gliomas a 10-year population-based study: a report from The Canadian Paediatric Brain Tumour Consortium (CPBTC). J Neurooncol. 2020;149(1):45–54. 32632896 10.1007/s11060-020-03568-8

[9] Unger JM, Barlow WE, Martin DP, et al Comparison of survival outcomes among cancer patients treated in and out of clinical trials. J Natl Cancer Inst. 2014;106(3):dju002. 24627276 10.1093/jnci/dju002 PMC3982777

[10] Van Houwelingen HC. Dynamic prediction by landmarking in event history analysis. Scand J Stat. 2007;34(1):70–85.

[11] Jansen MH, Veldhuijzen van Zanten SE, Sanchez Aliaga E, et al Survival prediction model of children with diffuse intrinsic pontine glioma based on clinical and radiological criteria. Neuro Oncol. 2015;17(1):160–166. 24903904 10.1093/neuonc/nou104 PMC4483042

[12] Leach JL, Roebker J, Schafer A, et al MR imaging features of diffuse intrinsic pontine glioma and relationship to overall survival: report from the International DIPG Registry. Neuro Oncol. 2020;22(11):1647–1657. 32506137 10.1093/neuonc/noaa140 PMC7690352

[13] Gallitto M, Lazarev S, Wasserman I, et al Role of radiation therapy in the management of diffuse intrinsic pontine glioma: a systematic review. Adv Radiat Oncol. 2019;4(3):520–531. 31360809 10.1016/j.adro.2019.03.009 PMC6639749

[14] Di Nunno V, Lombardi G, Simonelli M, et al The role of adjuvant chemotherapy in patients with H3K27 altered diffuse midline gliomas: a multicentric retrospective study. J Neurooncol. 2024;167(1):145–154. 38457090 10.1007/s11060-024-04589-3

[15] Lassman LP, Arjona VE. Pontine gliomas of childhood. Lancet. 1967;1(7496):913–915. 4164397 10.1016/s0140-6736(67)91485-7

[16] Ataç MS, Blaauw G. Radiotherapy in brain-stem gliomas in children. Clin Neurol Neurosurg. 1979;81(4):281–290. 233210 10.1016/0303-8467(79)90032-5

[17] Cooney T, Lane A, Bartels U, et al Contemporary survival endpoints: an International Diffuse Intrinsic Pontine Glioma Registry study. Neuro Oncol. 2017;19(9):1279–1280. 28821206 10.1093/neuonc/nox107 PMC5570207

[18] Hargrave D, Bartels U, Bouffet E. Diffuse brainstem glioma in children: critical review of clinical trials. Lancet Oncol. 2006;7(3):241–248. 16510333 10.1016/S1470-2045(06)70615-5

[19] Wolff JE, Rytting ME, Vats TS, et al Treatment of recurrent diffuse intrinsic pontine glioma: the MD Anderson Cancer Center experience. J Neurooncol. 2012;106(2):391–397. 21858608 10.1007/s11060-011-0677-3 PMC3990187

[20] MacDonald TJ, Aguilera D, Kramm CM. Treatment of high-grade glioma in children and adolescents. Neuro Oncol. 2011;13(10):1049–1058. 21784756 10.1093/neuonc/nor092 PMC3177659

[21] Kyriacou DN, Lewis RJ. Confounding by Indication in Clinical Research. JAMA. 2016;316(17):1818–1819. 27802529 10.1001/jama.2016.16435

[22] Bartlett AL, Lane A, Chaney B, et al Characteristics of children ≤36 months of age with DIPG: a report from the international DIPG registry. Neuro Oncol. 2022;24(12):2190–2199. 35552452 10.1093/neuonc/noac123 PMC9713498

[23] Broniscer A, Laningham FH, Sanders RP, et al Young age may predict a better outcome for children with diffuse pontine glioma. Cancer. 2008;113(3):566–572. 18484645 10.1002/cncr.23584

[24] Erker C, Lane A, Chaney B, et al Characteristics of patients ≥10 years of age with diffuse intrinsic pontine glioma: a report from the International DIPG/DMG Registry. Neuro Oncol. 2022;24(1):141–152. 34114629 10.1093/neuonc/noab140 PMC8730773

[25] Jones C, Karajannis MA, Jones DTW, et al Pediatric high-grade glioma: biologically and clinically in need of new thinking. Neuro Oncol. 2017;19(2):153–161. 27282398 10.1093/neuonc/now101 PMC5464243

[26] Pollack IF, Boyett JM, Yates AJ, et al; Children's Cancer Group. The influence of central review on outcome associations in childhood malignant gliomas: results from the CCG-945 experience. Neuro Oncol. 2003;5(3):197–207. 12816726 10.1215/S1152-8517-03-00009-7 PMC1920685

[27] Baugh J, Bartels U, Leach J, et al The international diffuse intrinsic pontine glioma registry: an infrastructure to accelerate collaborative research for an orphan disease. J Neurooncol. 2017;132(2):323–331. 28093680 10.1007/s11060-017-2372-5 PMC6343830

[28] Veldhuijzen van Zanten SEM, Baugh J, Chaney B, et al; members of the SIOPE DIPG Network. Development of the SIOPE DIPG network, registry and imaging repository: a collaborative effort to optimize research into a rare and lethal disease. J Neurooncol. 2017;132(2):255–266. 28110411 10.1007/s11060-016-2363-y PMC5378734

[29] Koschmann C, Al-Holou WN, Alonso MM, et al A road map for the treatment of pediatric diffuse midline glioma. Cancer Cell. 2024;42(1):1–5. 38039965 10.1016/j.ccell.2023.11.002 PMC11067690

[30] Aldape K, Brindle KM, Chesler L, et al Challenges to curing primary brain tumours. Nat Rev Clin Oncol. 2019;16(8):509–520. 30733593 10.1038/s41571-019-0177-5 PMC6650350

[31] Rojo de la Vega M. A holistic view of cancer. Cancer Cell. 2023;41(3):373. 36917947 10.1016/j.ccell.2023.02.011

[32] Majzner RG, Ramakrishna S, Yeom KW, et al GD2-CAR T cell therapy for H3K27M-mutated diffuse midline gliomas. Nature. 2022;603(7903):934–941. 35130560 10.1038/s41586-022-04489-4 PMC8967714

[33] Gállego Pérez-Larraya J, Garcia-Moure M, Labiano S, et al Oncolytic DNX-2401 virus for pediatric diffuse intrinsic pontine glioma. N Engl J Med. 2022;386(26):2471–2481. 35767439 10.1056/NEJMoa2202028

[34] Chi AS, Tarapore RS, Hall MD, et al Pediatric and adult H3 K27M-mutant diffuse midline glioma treated with the selective DRD2 antagonist ONC201. J Neurooncol. 2019;145(1):97–105. 31456142 10.1007/s11060-019-03271-3 PMC7241441

[35] Mueller S, Kline C, Stoller S, et al PNOC015: Repeated convection enhanced delivery (CED) of MTX110 (aqueous panobinostat) in children with newly diagnosed diffuse intrinsic pontine glioma (DIPG) [published online ahead of print, 2023 Jun 15]. Neuro Oncol. 2023;25(11):2074–2086. 37318058 10.1093/neuonc/noad105 PMC10628948

[36] Warren KE. Beyond the blood:brain barrier: the Importance of central nervous system (CNS) pharmacokinetics for the treatment of CNS tumors, including diffuse intrinsic pontine glioma. Front Oncol. 2018;8(1):239. 30018882 10.3389/fonc.2018.00239 PMC6037693

[37] Kim Y, Armstrong TS, Gilbert MR, Celiku O. A critical analysis of neuro-oncology clinical trials. Neuro Oncol. 2023;25(9):1658–1671. 36757281 10.1093/neuonc/noad036 PMC10484169

[38] Viele K, Berry S, Neuenschwander B, et al Use of historical control data for assessing treatment effects in clinical trials. Pharm Stat. 2014;13(1):41–54. 23913901 10.1002/pst.1589 PMC3951812

[39] Polley MC, Schwartz D, Karrison T, Dignam JJ. Leveraging external control data in the design and analysis of neuro-oncology trials: pearls and perils. Neuro Oncol. 2024;26(5):796–810. 38254183 10.1093/neuonc/noae005 PMC11066907

[40] Margol AS, Molinaro AM, Onar-Thomas A, et al Use of external control cohorts in pediatric brain tumor clinical trials. J Clin Oncol. 2024;42(12):1340–1343. 38394473 10.1200/JCO.23.01084

[41] Rahman R, Ventz S, McDunn J, et al Leveraging external data in the design and analysis of clinical trials in neuro-oncology. Lancet Oncol. 2021;22(10):e456–e465. 34592195 10.1016/S1470-2045(21)00488-5 PMC8893120

[42] Patil N, Kelly ME, Yeboa DN, et al Epidemiology of brainstem high-grade gliomas in children and adolescents in the United States, 2000-2017. Neuro Oncol. 2021;23(6):990–998. 33346835 10.1093/neuonc/noaa295 PMC8168816

[43] Tejada S, Aquilina K, Goodden J, et al Biopsy in diffuse pontine gliomas: expert neurosurgeon opinion-a survey from the SIOPE brain tumor group. Childs Nerv Syst. 2020;36(4):705–711. 32020269 10.1007/s00381-020-04523-8

