# Supplementary material for: Treatment-related survival patterns in diffuse intrinsic pontine glioma using a historical cohort: A report from the European Society for Pediatric Oncology DIPG/DMG Registry
Source: Neurooncol Adv. 2024 Sep 10;6(1):vdae155. doi: 10.1093/noajnl/vdae155 (PMC11582646; doi:10.1093/noajnl/vdae155)
Supplement: vdae155_suppl_Supplementary_Materials [file vdae155_suppl_Supplementary_Materials.docx]

Supplementary Table 1. Systemic therapy regimens (n=409).

| Regimen | *n* (%) |
| --- | --- |
| HIT-HGG-2007 | 145 (35.5) |
| HIT-GBM-D | 67 (16.4) |
| HIT-GBM-C | 36 (8.8) |
| HIT-GBM-B | 11 (2.7) |
| Temozolomide | 10 (2.4) |
| HIT-GBM-A | 6 (1.5) |
| Other chemotherapy | 6 (1.5) |
| Nimotuzumab | 3 (0.7) |
| Temozolomide+Nimotuzumab | 3 (0.7) |
| Erlotinib | 2 (0.5) |
| Sirolimus | 2 (0.5) |
| Vincristine | 2 (0.5) |
| Vinorelbine+Nimotuzumab | 2 (0.5) |
| Everolimus | 1 (0.2) |
| Methotrexate | 1 (0.2) |
| Temozolomide+Vinorelbine | 1 (0.2) |
| Temozolomide+Valproate | 1 (0.2) |
| N/A | 110 (26.9) |
|  |  |

Supplementary Table 2a. Number of events from diagnosis

|  |  |  |  |
| --- | --- | --- | --- |
| Status | No treatment | Radiotherapy | Radiochemotherapy |
| Alive | 0 | 1 | 11 |
| Deceased | 20 | 89 | 288 |
| Total | 20 | 90 | 299 |

Supplementary Table 2b. Number of events from relapse

|  |  |  |  |
| --- | --- | --- | --- |
| Status | No treatment | Chemotherapy | Reirradiation, +/-chemotherapy |
| Alive | 4 | 0 | 2 |
| Deceased | 96 | 191 | 49 |
| Total | 100 | 191 | 51 |
